# c-di-GMP Inhibits Early Sporulation in Clostridioides difficile

**DOI:** 10.1128/msphere.00919-21

**Published:** 2021-12-08

**Authors:** Adrianne N. Edwards, Caitlin L. Willams, Nivedita Pareek, Shonna M. McBride, Rita Tamayo

**Affiliations:** a Department of Microbiology and Immunology, Emory University School of Medicine, Emory Antibiotic Resistance Center, Atlanta, Georgia, USA; b Department of Microbiology and Immunology, University of North Carolina—Chapel Hill, Chapel Hill, North Carolina, USA; University of Iowa

**Keywords:** *Clostridioides difficile*, *Clostridium difficile*, sporulation, spore, cyclic diguanylate, c-di-GMP, anaerobe, cyclic diguanylate synthase

## Abstract

The formation of dormant spores is essential for the anaerobic pathogen Clostridioides difficile to survive outside the host gastrointestinal tract. The regulatory pathways and environmental signals that initiate C. difficile spore formation within the host are not well understood. One second-messenger signaling molecule, cyclic diguanylate (c-di-GMP), modulates several physiological processes important for C. difficile pathogenesis and colonization, but the impact of c-di-GMP on sporulation is unknown. In this study, we investigated the contribution of c-di-GMP to C. difficile sporulation. The overexpression of a gene encoding a diguanylate cyclase, *dccA*, decreased the sporulation frequency and early sporulation gene transcription in both the epidemic R20291 and historical 630Δ*erm* strains. The expression of a *dccA* allele encoding a catalytically inactive DccA that is unable to synthesize c-di-GMP no longer inhibited sporulation, indicating that the accumulation of intracellular c-di-GMP reduces C. difficile sporulation. A null mutation in *dccA* slightly increased sporulation in R20291 and slightly decreased sporulation in 630Δ*erm*, suggesting that DccA contributes to the intracellular pool of c-di-GMP in a strain-dependent manner. However, these data were highly variable, underscoring the complex regulation involved in modulating intracellular c-di-GMP concentrations. Finally, the overexpression of *dccA* in known sporulation mutants revealed that c-di-GMP is likely signaling through an unidentified regulatory pathway to control early sporulation events in C. difficile. c-di-GMP-dependent regulation of C. difficile sporulation may represent an unexplored avenue of potential environmental and intracellular signaling that contributes to the complex regulation of sporulation initiation.

**IMPORTANCE** Many bacterial organisms utilize the small signaling molecule cyclic diguanylate (c-di-GMP) to regulate important physiological processes, including motility, toxin production, biofilm formation, and colonization. c-di-GMP inhibits motility and toxin production and promotes biofilm formation and colonization in the anaerobic, gastrointestinal pathogen Clostridioides difficile. However, the impact of c-di-GMP on C. difficile spore formation, a critical step in this pathogen’s life cycle, is unknown. Here, we demonstrate that c-di-GMP negatively impacts sporulation in two clinically relevant C. difficile strains, the epidemic strain R20291 and the historical strain 630Δ*erm*. The pathway through which c-di-GMP controls sporulation was investigated, and our results suggest that c-di-GMP is likely signaling through an unidentified regulatory pathway to control C. difficile sporulation. This work implicates c-di-GMP metabolism as a mechanism to integrate environmental and intracellular cues through c-di-GMP levels to influence C. difficile sporulation.

## INTRODUCTION

Nucleotide second messengers, such as the nearly ubiquitous cyclic diguanylate (c-di-GMP), serve as central intracellular signaling molecules in bacteria. c-di-GMP promotes the switch between a planktonic, motile stage and a sessile, surface-associated lifestyle and controls virulence factor production in numerous pathogenic and nonpathogenic bacteria. c-di-GMP is synthesized from GTP by diguanylate cyclases (DGCs) (synthases), which contain the conserved catalytic GGDEF motif (1). Phosphodiesterases (PDEs) (hydrolases) containing either the EAL or HD-GYP domain degrade c-di-GMP to pGpG or GMP, respectively (2–5). Often, DGC and PDE proteins contain additional sensory or regulatory domains that potentially control enzymatic activity, suggesting that environmental and bacterial cues influence the intracellular concentration of c-di-GMP (6). Most Gram-negative bacteria encode numerous DGC and PDE proteins, resulting in complex c-di-GMP metabolic pathways, while Gram-positive bacteria often contain more modest numbers of DGC and PDE proteins (7–9). However, the gastrointestinal pathogen Clostridioides difficile encodes many c-di-GMP metabolic proteins; 37 genes encoding GGDEF and/or EAL domains were identified in the historical 630 strain (10, 11). Many of the C. difficile c-di-GMP metabolic proteins have been demonstrated to be enzymatically active (10, 12), suggesting that the regulation of c-di-GMP metabolism in C. difficile is physiologically important and tightly controlled. High intracellular concentrations of c-di-GMP have been demonstrated to inhibit C. difficile motility and toxin production while promoting cell aggregation, biofilm formation, and colonization (13–20).

C. difficile is an obligate anaerobe and relies on the formation of a dormant spore for long-term persistence outside the host and transmission to new hosts. Spore formation in all endospore-forming bacteria, including C. difficile, is initiated by the highly conserved transcriptional regulator Spo0A (21, 22). Spo0A activity is tightly controlled by phosphorylation through a large regulatory network of kinases, phosphatases, and additional regulators (23, 24). Once phosphorylated, Spo0A∼P activates the expression of early sporulation genes, triggering sporulation (25; M. A. DiCandia and S. M. McBride, unpublished data). However, many of the regulatory proteins and pathways that control early sporulation events in the model organism Bacillus subtilis are not conserved in C. difficile (26, 27). Although recent progress has uncovered several important sporulation regulatory factors in C. difficile (reviewed in reference 28), the environmental cues and regulatory pathways that control Spo0A activation are largely unknown.

Environmental conditions and nutrient availability likely trigger C. difficile spore formation within the host gastrointestinal tract. Two global transcriptional regulators, CodY and CcpA, control sporulation initiation in C. difficile in response to nutrient availability. CodY represses target gene expression when GTP and branched-chain amino acids are abundant, and CcpA activates or represses target gene transcription based on carbohydrate availability (29, 30). Mutations in either CodY or CcpA result in increased sporulation frequencies (30, 31). Some sporulation genes serve as direct targets for CodY and CcpA regulation, but the molecular mechanisms are not delineated (29–31). Additionally, the uptake of peptides, a critical nutrient source for C. difficile (32), by the Opp and App oligopeptide permeases affects the timing of sporulation, as *opp* and *app* inactivation significantly increases sporulation frequencies (33). It is reasonable to consider that other global signaling systems in C. difficile link nutrient availability and/or other environmental conditions to the decision to initiate sporulation.

In this study, we examined the impact that c-di-GMP has on C. difficile sporulation. We show that the overexpression of *dccA*, a gene encoding a DGC, resulted in decreased sporulation in two important C. difficile strains. The conserved catalytic motif GGDEF was required for DccA-dependent inhibition of C. difficile spore formation, indicating that c-di-GMP metabolic activity is responsible for this phenotype. Finally, we provide evidence that c-di-GMP does not depend on signaling through several known sporulation factors, suggesting that c-di-GMP influences sporulation through an unidentified pathway.

## RESULTS

### Overexpression of a diguanylate cyclase reduces C. difficile sporulation frequency.

As c-di-GMP affects many physiological processes in C. difficile, we hypothesized that C. difficile sporulation is also influenced by c-di-GMP. To test this hypothesis, *dccA*, which encodes a DGC in C. difficile (10, 13), was overexpressed on a multicopy plasmid using the nisin-inducible *cpr* promoter (13, 33, 34) in two different C. difficile backgrounds. We included R20291, which is a clinically prevalent epidemic strain, and 630Δ*erm*, which is a spontaneous erythromycin-sensitive derivative of 630, a clinical isolate that has served as a long-term laboratory model strain (35, 36). Of note, the amino acid sequences of DccA from R20291 and 630Δ*erm* are 100% identical. To assess sporulation frequency, we performed ethanol-resistant sporulation assays in these strains after 24 h of growth (H_24_) on 70:30 sporulation agar.

**FIG 1:**
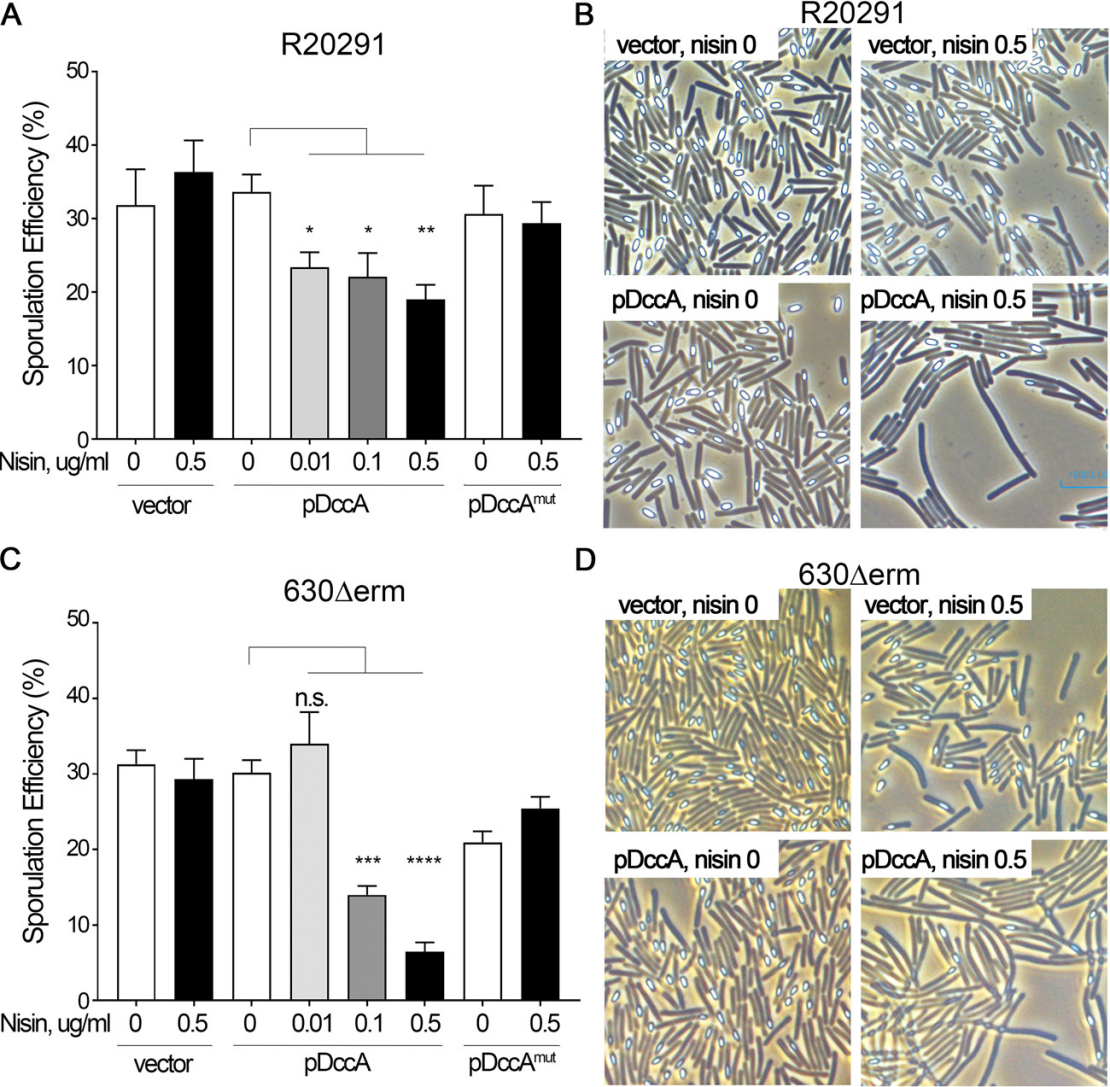
Overexpression of *dccA* inhibits sporulation in R20291 and 630Δ*erm* and is dependent upon a functional cyclic diguanylate (GGDEF) domain. (A and B) Ethanol-resistant sporulation assays (A) and representative phase-contrast micrographs (B) of R20291 pMC211 (RT526), R20291 pDccA (RT527), and R20291 pDccA^mut^ (RT539) grown on 70:30 sporulation agar supplemented with 2 μg/ml thiamphenicol and 0 to 0.5 μg/ml nisin, as indicated, at H_24_. (C and D) Ethanol-resistant sporulation assays (C) and representative phase-contrast micrographs (D) of 630Δ*erm* pMC211 (RT762), 630Δ*erm* pDccA (RT763), and 630Δ*erm* pDccA^mut^ (RT764) grown on 70:30 sporulation agar supplemented with 2 μg/ml thiamphenicol and 0 to 0.5 μg/ml nisin, as indicated, at H_24_. The means and standard errors of the means from at least three biological replicates are shown. n.s., not significant; *, *P* < 0.05; **, *P* < 0.01; ***, *P* < 0.001; ****, *P* < 0.0001 (by one-way analysis of variance [ANOVA] with Dunnett’s posttest comparing values to that of uninduced WT pDccA).

When a plasmid copy of *dccA* (pDccA) was overexpressed from the nisin-inducible promoter in the R20291 background, the sporulation frequency significantly decreased from 33.7% in the absence of nisin to 19% in the presence of 0.5 μg/ml nisin (Fig. 1A), suggesting that the overexpression of *dccA* and, presumably, high intracellular levels of c-di-GMP inhibit C. difficile sporulation. To assess whether the decrease in the sporulation frequency was due to the production of c-di-GMP by DccA, we overexpressed a *dccA* allele encoding a mutated GGDEF domain (AADEF) that is unable to synthesize c-di-GMP (pDccA^mut^) (13). Here, even at the highest expression level of *dccA* (0.5 μg/ml nisin), the sporulation efficiency remained unaffected, indicating that the DccA-dependent reduction in the sporulation frequency is due to DccA’s diguanylate cyclase activity. We also visualized sporulation in these strains using phase-contrast microscopy, in which spores appear phase bright and vegetative cells are phase-dark rods. When *dccA* was overexpressed in R20291, not only were fewer spores visible, but this strain also formed long chains (Fig. 1B; discussed below). The reduced sporulation frequency and cell morphology phenotypes are dependent on increased c-di-GMP concentrations as these effects were not seen in the R20291 strain overexpressing pDccA^mut^ (data not shown).

When *dccA* was overexpressed in the 630Δ*erm* background, we observed an ∼4-fold decrease in the sporulation efficiency (29.7% in the absence of nisin to 8.5% with 0.5 μg/ml nisin) (Fig. 1C). This reduction in sporulation frequency in the 630Δ*erm* pDccA strain was dose dependent, with the lowest sporulation frequency occurring at the highest expression level of *dccA* (0.5 μg/ml nisin). Again, the overexpression of the *dccA*^mut^ allele resulted in wild-type (WT) levels of sporulation, indicating that the ability of DccA to repress sporulation relies on a functional GGDEF domain and its diguanylate cyclase activity. Similar to R20291, the overexpression of *dccA* in the 630Δ*erm* background resulted in fewer spores and a change in cell morphology when observed by phase-contrast microscopy, and these phenotypes were also dependent on a functional DccA GGDEF domain (Fig. 1D and data not shown).

**FIG 2:**
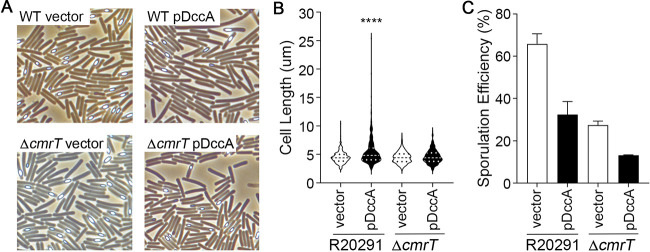
c-di-GMP-dependent cell chaining is dependent on CmrT. (A and B) Representative phase-contrast micrographs (A) and quantification of cell length using ImageJ (B) of R20291 pMC211 (RT526), R20291 pDccA (RT527), R20291 *cmrT* pMC211 (RT2283), and R20291 *cmrT* pDccA (RT2284) grown on 70:30 sporulation agar supplemented with 2 μg/ml thiamphenicol and 0.5 μg/m nisin at H_24_. For panel B, cell lengths of 100 bacterial cells from 3 biological replicates were measured (*n* = 300). The violin plot shows distributions, medians, and quartiles. ****, *P* < 0.0001 (by one-way ANOVA and Tukey’s posttest compared to all other strains). (C) Ethanol-resistant sporulation assays of R20291 pMC211 (RT526), R20291 pDccA (RT527), R20291 *cmrT* pMC211 (RT2283), and R20291 *cmrT* pDccA (RT2284) grown on 70:30 sporulation agar supplemented with 2 μg/ml thiamphenicol and 0.5 μg/m nisin at H_24_. The means and standard errors of the means from at least three biological replicates are shown. *, *P* < 0.05; **, *P* < 0.01; ***, *P* < 0.001; ****, *P* < 0.0001 (by one-way ANOVA and Tukey’s posttest).

As noted above, the overexpression of *dccA* resulted in dramatic cell morphology changes in R20291 and 630Δ*erm* that are dependent on increased intracellular concentrations of c-di-GMP (Fig. 1B and D). This change in cell morphology with *dccA* overexpression was expected and is likely due to the c-di-GMP-dependent increase in the expression of *cmrRST*, which encodes an atypical signal transduction system that regulates C. difficile colony morphology and motility (37, 38). CmrT promotes bundled cell chaining. To determine whether CmrT is responsible for the formation of long chains at high c-di-GMP concentrations, we overexpressed *dccA* in the R20291 *cmrT* background. As predicted, the elongated cells observed at high c-di-GMP concentrations are no longer present in the *cmrT* mutant (Fig. 2A and B), indicating that c-di-GMP promotes cell chaining through the activation of *cmrT* expression. CmrT is not involved in the c-di-GMP-dependent inhibition of sporulation, as the sporulation frequency of the *cmrT* mutant is decreased ∼2-fold, similar to the R20291 parent strain, when *dccA* is overexpressed (Fig. 2C).

### Overexpression of a cyclic diguanylate decreases sporulation-specific gene expression.

**FIG 3:**
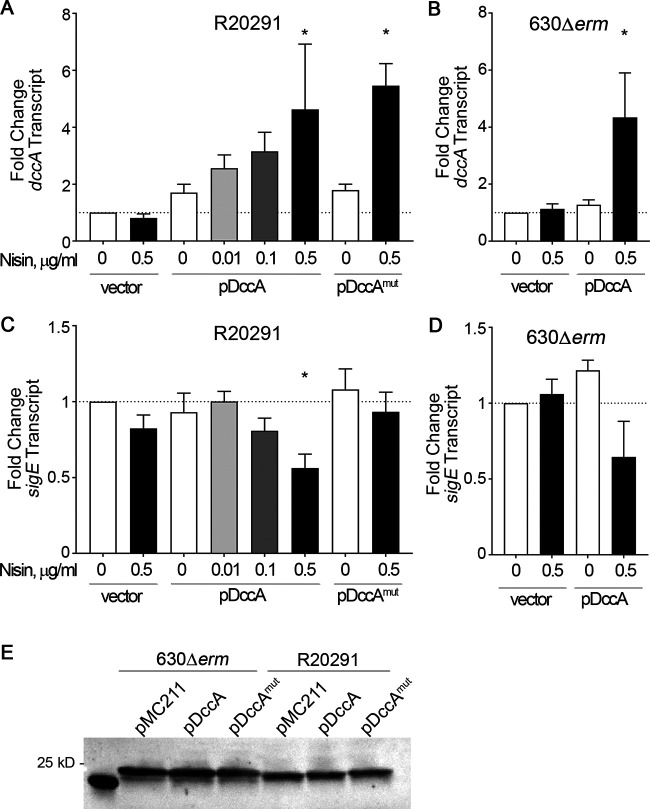
Overexpression of *dccA* decreases Spo0A-dependent gene expression. (A and C) qRT-PCR analyses of *dccA* (A) and *sigE* (C) transcript levels in R20291 pMC211 (RT526), R20291 pDccA (RT527), and R20291 pDccA^mut^ (RT539) grown on 70:30 sporulation agar supplemented with 2 μg/ml thiamphenicol and 0 to 0.5 μg/ml nisin, as indicated, at H_12_. (B and D) qRT-PCR analyses of *dccA* (B) and *sigE* (D) transcript levels in 630Δ*erm* pMC211 (RT762) and 630Δ*erm* pDccA (RT763) grown on 70:30 sporulation agar supplemented with 2 μg/ml thiamphenicol in the absence or presence of 0.5 μg/ml nisin at H_12_. The means and standard errors of the means from at least three biological replicates are shown. *, *P* < 0.05 (by one-way ANOVA with Dunnett’s posttest comparing values to that of the uninduced WT vector). (E) Anti-Spo0A Western blot analysis of 630Δ*erm* pMC211 (RT762), 630Δ*erm* pDccA (RT763), 630Δ*erm* pDccA^mut^ (RT764), R20291 pMC211 (RT526), R20291 pDccA (RT527), and R20291 pDccA^mut^ (RT539) grown on 70:30 sporulation agar supplemented with 2 μg/ml thiamphenicol and 0.5 μg/ml nisin at H_12_.

To ensure that *dccA* and *dccA*^mut^ transcription are activated in a dose-dependent manner with increasing concentrations of nisin, we measured *dccA* transcript levels using quantitative reverse transcription-PCR (qRT-PCR). Cells were harvested after 12 h of growth on 70:30 sporulation agar, which marks early stationary phase and the onset of sporulation (39). As expected, *dccA* and *dccA*^mut^ transcript levels were increased ∼4- to 6-fold in both R20291 and 630Δ*erm* in the presence of 0.5 μg/ml nisin (Fig. 3A and B), showing that *dccA* and *dccA*^mut^ expression levels are consistent in both backgrounds. To better understand the effect of c-di-GMP on early sporulation events in C. difficile, we measured the steady-state transcript levels of an early sporulation-specific gene, *sigE* (*spoIIG*), using qRT-PCR. The transcription of *sigE* is dependent on active, phosphorylated Spo0A (40). The relative expression levels of *sigE* in both R20291 pDccA and 630Δ*erm* pDccA grown on 0.5 μg/ml nisin were decreased ∼2-fold compared to the levels in their respective parent strains (Fig. 3C and D). The decreased *sigE* transcript levels in R20291 pDccA were dependent upon a functional GGDEF domain as *sigE* transcript levels were unchanged when *dccA*^mut^ was overexpressed in R20291 (Fig. 3C).

To further assess how c-di-GMP impacts early sporulation events, we measured the transcript levels of additional early-sporulation-specific genes, including *spo0A*, *murG* (a Spo0A-dependent gene), *sigF* (encodes the early sporulation-specific sigma factor that is expressed in the forespore compartment), *gpr* (a SigF-dependent gene), and *spoIID* (a SigE-dependent gene). The transcript levels of early sporulation genes were minimally affected in the R20291 background; however, with the exception of *spo0A*, there was a significant decrease in early sporulation gene expression when *dccA* was overexpressed in the 630Δ*erm* background (Table 1). These results may reflect the stronger impact on sporulation that *dccA* overexpression has on 630Δ*erm* than on R20291. Mirroring *spo0A* transcript levels in R20291 and 630Δ*erm*, Spo0A protein levels were unchanged regardless of *dccA* overexpression (Fig. 3E). The lack of changes in *spo0A* transcript and protein levels is expected given that Spo0A activity is controlled by posttranslational phosphorylation. Furthermore, Spo0A autoregulation has not been observed in other C. difficile sporulation mutants, including the hypersporulating *opp app* and oligosporogenous *rstA* mutants (33, 39), despite affecting Spo0A activity and Spo0A-dependent gene expression. These data indicate that the overexpression of *dccA* reduces early sporulation gene expression and confirm that the increased production of c-di-GMP is responsible for reduced sporulation in C. difficile.

**TABLE 1:** Effect of *dccA* overexpression on sporulation-specific gene expression in R20291 and 630Δ*erm*

Transcript^*a*^	Strain^*b*^	Mean fold change ± SD^*c*^		
		pMC211	pDccA	pDccA^mut^
*spo0A*	R20291	0.91 ± 0.12	0.96 ± 0.27	0.77 ± 0.11
	630Δ*erm*	0.79 ± 0.07	0.96 ± 0.16	ND

*murG * (Spo0A dependent)	R20291	0.85 ± 0.08	0.72 ± 0.08	0.79 ± 0.06
	630Δ*erm*	1.19 ± 0.15	**0.60 ± 0.05**	ND

*sigF*	R20291	0.97 ± 0.20	1.15 ± 0.38	0.95 ± 0.23
	630Δ*erm*	0.74 ± 0.10	0.47 ± 0.11	ND

*gpr * (SigF dependent)	R20291	0.82 ± 0.10	0.6 ± 0.17	0.9 ± 0.10
	630Δ*erm*	1.16 ± 0.19	**0.44 ± 0.15**	ND

*spoIID * (SigE dependent)	R20291	0.73 ± 0.14	0.48 ± 0.08	0.67 ± 0.13
	630Δ*erm*	1.32 ± 0.13	**0.72 ± 0.06**	ND

a All mean fold change values are relative to the respective parent strain containing pMC211 grown in the presence of no nisin.

b Strains were harvested at H_12_ from 70:30 sporulation agar supplemented with 2 μg/ml thiamphenicol and 0.5 μg/ml nisin.

c Boldface type indicates a *P* value of ≤0.05 by Student’s *t* test (630Δ*erm* background). There was no statistically significant data in the R20291 background as determined by one-way ANOVA followed by Dunnett’s multiple-comparison test (R20291 background). ND, not determined.

### Overexpression of *dccA* increases c-di-GMP-dependent gene expression in a dose-dependent manner on 70:30 sporulation agar.

**FIG 4:**
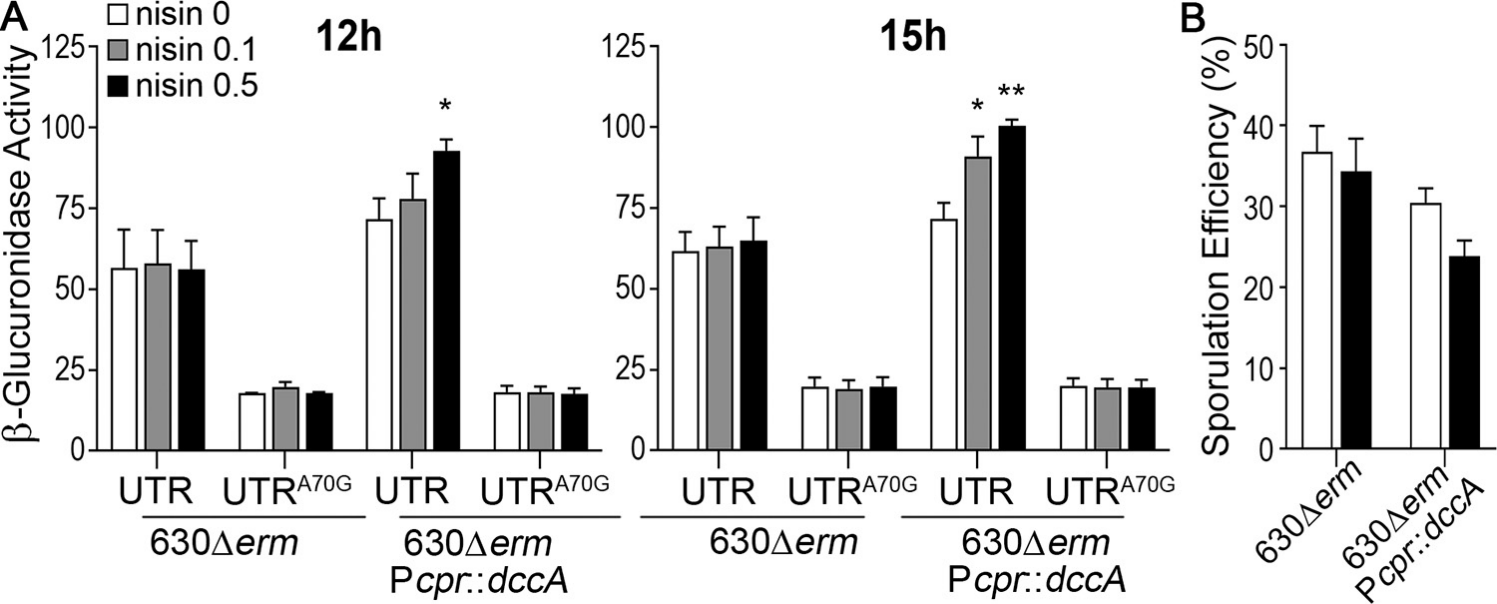
Overexpression of *dccA* results in a dose-dependent increase in c-di-GMP-dependent gene expression. (A) β-Glucuronidase reporter activity in 630Δ*erm* pP*_pilA1_*-UTR-*gusA* (RT1050), 630Δ*erm* pP*_pilA1_*-UTR^A70G^-*gusA* (RT1051), 630Δ*erm*::P*_cprA_-dccA* pP*_pilA1_*-UTR-*gusA* (RT1054), and 630Δ*erm*::P*_cprA_-dccA* pP*_pilA1_*-UTR^A70G^-*gusA* (RT1055) grown on 70:30 sporulation agar supplemented with 2 μg/ml thiamphenicol in the absence or presence of 0.1 μg/ml or 0.5 μg/ml nisin at H_12_ and H_15_. (B) Ethanol-resistant sporulation assays of 630Δ*erm* and 630Δ*erm*::P*_cprA_-dccA* (RT993) grown on 70:30 sporulation agar in the presence or absence of 0.5 μg/ml nisin at H_24_. The means and standard errors of the means from three biological replicates are shown. *, *P* < 0.05; **, *P* < 0.01 (by one-way ANOVA and Dunnett’s posttest comparing values to that of the WT vector, with 0.5 μg/ml nisin).

We previously utilized high-performance liquid chromatography–tandem mass spectrometry (HPLC-MS/MS) to measure the intracellular concentration of c-di-GMP when *dccA* is overexpressed from the nisin-inducible promoter (13). To assess the relative increase in intracellular c-di-GMP with *dccA* induction during growth on 70:30 sporulation agar, we employed the previously described reporter controlled by the regulatory region of the *pilA1* locus: the *pilA1* promoter (P*_pilA_*) and the 5′ untranslated region (UTR) containing a c-di-GMP riboswitch (41). c-di-GMP directly and positively regulates *pilA* transcription via the c-di-GMP-responsive riboswitch located in the *pilA* 5′ UTR (16). This P*_pilA1_*-UTR-*gusA* reporter was introduced into 630Δ*erm* with chromosomal nisin-inducible *dccA* (41). As a control, we also utilized a P*_pilA1_*-UTR^A70G^-*gusA* reporter where a single nucleotide substitution renders the riboswitch unresponsive to c-di-GMP (16, 41). We measured P*_pilA1_*-UTR-*gusA* reporter activity after growth on 70:30 sporulation agar in the presence of no nisin or 0.1 μg/ml or 0.5 μg/ml nisin at H_12_ and H_15_. These time points represent early stationary phase, at the point where the sporulation regulatory cascade is initiated and sporulation-specific gene expression is active. Without induction, basal expression from the chromosomally encoded P*_cpr_-dccA* construct resulted in a slight increase (∼1.2-fold) in β-glucuronidase activity compared to that of the parent strain at both time points (Fig. 4A). We observed ∼1.4-fold and ∼1.6-fold increases in β-glucuronidase activity in the presence of 0.1 μg/ml and 0.5 μg/ml nisin, respectively, compared to the parental control. As expected, the c-di-GMP-blind P*_pilA1_*-UTR^A70G^-*gusA* reporter exhibited significantly reduced and constitutive activity under all tested conditions (Fig. 4A).

Because *dccA* is overexpressed from the chromosome in these reporter assays and not from a plasmid as we did in our previous experiments, we assessed the impact on sporulation frequency when *dccA* expression was induced from the chromosome. The sporulation frequency of 630Δ*erm*::P*_cprA_-dccA* grown on 70:30 sporulation agar plates supplemented with 0.5 μg/ml nisin was ∼1.44-fold decreased compared to the 630Δ*erm* parent (Fig. 4B). As anticipated, the decrease in the sporulation efficiency was muted compared to *dccA* overexpression on the plasmid, likely due to differences in copy numbers. However, increasing the intracellular c-di-GMP concentration through *dccA* overexpression impacts both c-di-GMP-specific gene regulation and sporulation frequency. Altogether, these data confirm that c-di-GMP levels are modestly induced to physiologically relevant concentrations that affect c-di-GMP-dependent physiological processes under these conditions.

### Deletion of *dccA* results in variable sporulation frequencies in R20291 and 630Δ*erm*.

Sporulation may be impacted by one or a subset of c-di-GMP metabolic enzymes. C. difficile 630 encodes 37 proteins containing GGDEF and/or EAL domains, and R20291 encodes 31 (10, 11, 35). The overexpression of *dccA* bypasses the endogenous control of c-di-GMP. To better understand the c-di-GMP regulatory mechanism, we next asked whether a null mutation in *dccA* alone affects the C. difficile sporulation frequency. To test this hypothesis, we employed the pseudo-suicide vectors pMSR and pMSR0 (tailored for the 630 and R20291 backgrounds, respectively), which take advantage of allelic exchange and an inducible C. difficile toxin-antitoxin system to create a markerless gene deletion (42).

**FIG 5:**
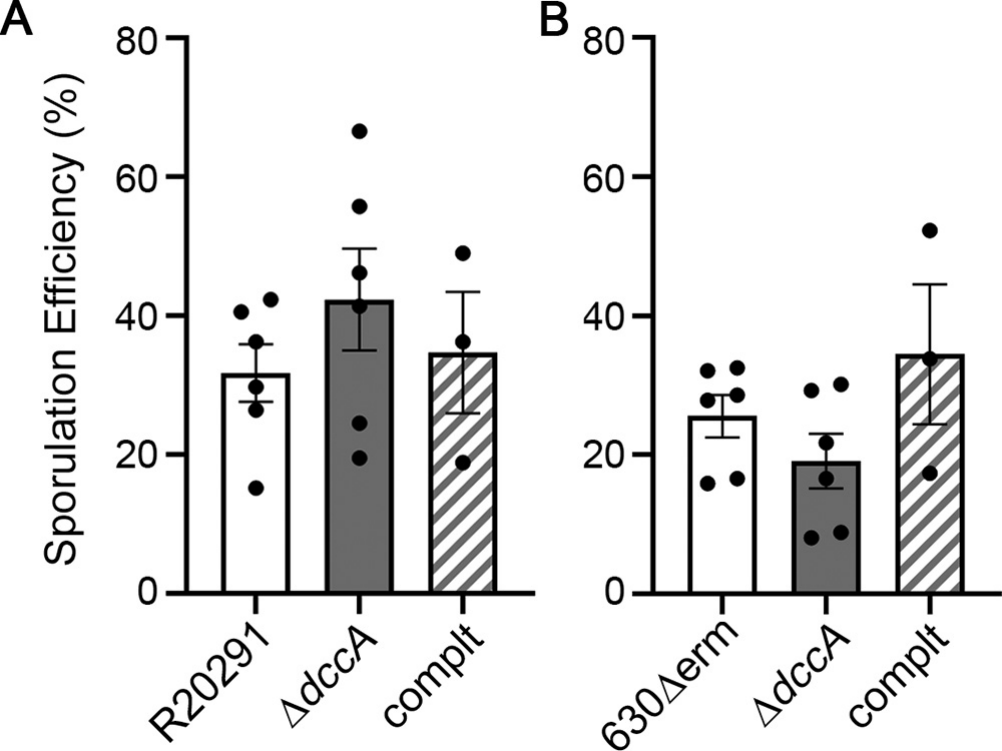
A null *dccA* mutation variably affects R20291 and 630Δ*erm* sporulation. Shown are data from ethanol-resistant sporulation assays of R20291, R20291 Δ*dccA* (RT2656), and R20291 Δ*dccA* Tn*916*::P*_CD1421_*-*dccA* (MC1961) (A) and 630Δ*erm*, 630Δ*erm* Δ*dccA* (RT2703), and 630Δ*erm* Δ*dccA* Tn*916*::P*_CD1421_*-*dccA* (MC1960) (B) grown on 70:30 sporulation agar at H_24_. The means and standard errors of the means from at least three biological replicates are shown. No significant differences were determined by one-way ANOVA. complt, complemented strain.

The R20291 Δ*dccA* mutant produced slightly more spores than R20291 (42.3% in R20291 Δ*dccA* versus 31.7% in the isogenic parent) (Fig. 5A). However, the sporulation frequencies were highly variable, and the differences between strains were not statistically significant. The R20291 Δ*dccA* mutant was complemented by integrating the *dccA* gene into the chromosome using the conjugative transposon Tn*916*. *dccA* is the second gene in a two-gene operon in R20291 and 630Δ*erm*, and the promoter of the upstream gene, *CD1421*, was used to drive *dccA* transcription in the complementation construct. The sporulation frequency of R20291 Δ*dccA* Tn*916*::P*_CD1421_*-*dccA* was reduced (34.7%) compared to that of the *dccA* mutant, but again, the data were variable and not statistically significant.

The 630Δ*erm* Δ*dccA* mutant exhibited a slightly reduced sporulation frequency compared to the 630Δ*erm* parent (19.1% in the *dccA* mutant versus 25.6% in the parent strain) (Fig. 5B), an opposite trend compared to that of the R20291 Δ*dccA* mutant. The complemented strain 630Δ*erm* Δ*dccA* Tn*916*::P*_CD1421_*-*dccA* showed an increased sporulation frequency (34.4%). But, as in the R20291 background, the sporulation frequencies were variable and did not achieve statistical significance. Altogether, these data suggest that c-di-GMP synthesis by DccA does not greatly and/or consistently contribute to sporulation initiation under the conditions tested or that other c-di-GMP metabolic enzymes are redundant with or compensate for the loss of DccA.

### c-di-GMP likely does not signal through known C. difficile sporulation factors.

**FIG 6:**
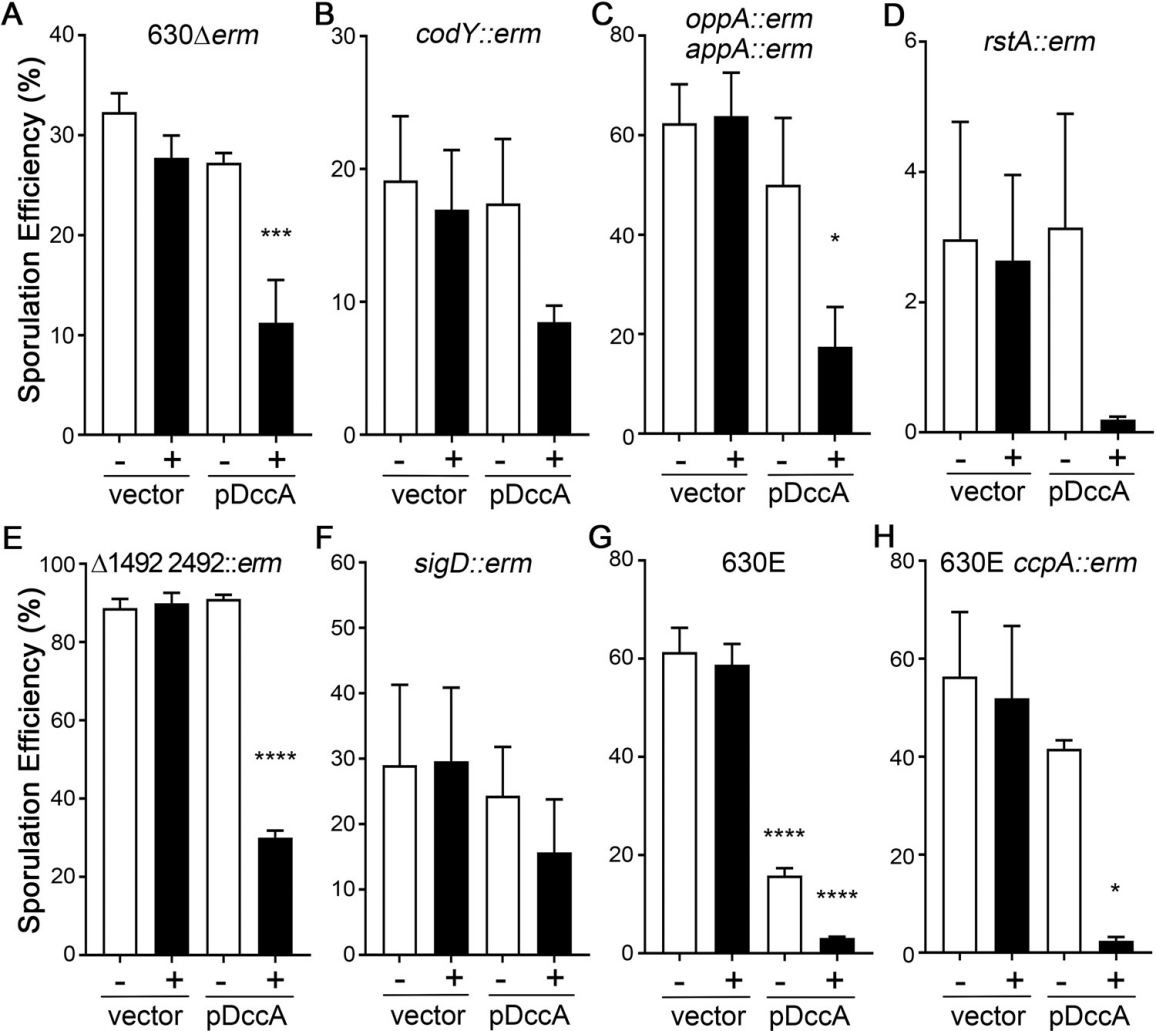
c-di-GMP does not inhibit sporulation through known sporulation factors. Shown are data from ethanol-resistant sporulation assays of 630Δ*erm* pMC211 (RT762) and 630Δ*erm* pDccA (RT763) (A), 630Δ*erm codY*::*erm* pMC211 (MC947) and 630Δ*erm codY*::*erm* pDccA (MC948) (B), 630Δ*erm oppB*::*erm appA*::*erm* pMC211 (MC924) and 630Δ*erm oppB*::*erm appA*::*erm* pDccA (MC925) (C), 630Δ*erm rstA*::*erm* pMC211 (MC926) and 630Δ*erm rstA*::*erm* pDccA (MC927) (D), 630Δ*erm* Δ*CD1492 CD2492*::*erm* pMC211 (MC928) and 630Δ*erm* Δ*CD1492 CD2492*::*erm* pDccA (MC929) (E), 630Δ*erm sigD*::*erm* pMC211 (MC864) and 630Δ*erm sigD*::*erm* pDccA (MC865) (F), 630E pMC211 (MC943) and 630E pDccA (MC944) (G), and 630E *ccpA*::*erm* pMC211 (MC945) and 630E *ccpA*::*erm* pDccA (MC946) (H) grown on 70:30 sporulation agar supplemented with 2 μg/ml thiamphenicol in the absence or presence of 0.5 μg/ml nisin, as indicated, at H_24_. The means and standard errors of the means from at least three biological replicates are shown. *, *P* < 0.05; ***, *P* < 0.001; ****, *P* < 0.0001 (by one-way ANOVA with Dunnett’s posttest comparing values to those of the respective parent strains with an uninduced vector). Note that the *y* axes for each panel vary depending on the sporulation frequency of the strain tested.

To attempt to identify the regulatory pathway(s) through which c-di-GMP influences sporulation in C. difficile, we overexpressed *dccA* in several well-studied 630Δ*erm* sporulation mutants and performed ethanol-resistant sporulation assays after 24 h of growth on 70:30 sporulation agar. We included the parent 630Δ*erm* strain overexpressing *dccA* in the absence and presence of 0.5 μg/ml nisin as a reference in these experiments (Fig. 6A).

First, we assessed the effect of *dccA* overexpression on 630Δ*erm codY* and 630Δ*erm opp app* mutants. CodY is a global transcriptional regulator that binds to target DNA at high intracellular concentrations of the effectors GTP and branched-chain amino acids (BCAAs) (43). The loss of GTP and BCAA binding changes the conformation of CodY, the differential expression of metabolic pathways, and other physiological processes, including toxin production and sporulation (29, 31, 44, 45). The *opp* and *app* operons each encode oligopeptide permeases that import small, heterogeneous peptides into the cell (46, 47). The inactivation of these permeases in C. difficile results in significantly increased sporulation, suggesting that limited nutrient uptake triggers sporulation (33). The regulatory pathways and molecular mechanisms by which CodY, Opp, and App affect sporulation are not fully understood, although null mutations in these loci affect sporulation timing and result in increased sporulation frequencies (31, 33). The sporulation frequencies of the *codY* mutant and the *opp app* double mutant decreased when *dccA* was overexpressed using 0.5 μg/ml nisin (2-fold and 3.7-fold, respectively, compared to each strain’s vector controls grown in 0.5 μg/ml nisin) (Fig. 6B and C). These results indicate that c-di-GMP does not require CodY or the Opp and App oligopeptide permeases to inhibit C. difficile sporulation.

Next, we ascertained whether RstA or the phosphotransfer proteins CD1492 and CD2492 are part of the regulatory pathway that c-di-GMP employs to control sporulation. RstA is a multifunctional protein that serves as a transcriptional regulator and, through a separate domain, positively influences C. difficile sporulation initiation via an unknown mechanism (39). The overexpression of *dccA* in the *rstA* background resulted in an ∼13-fold-decreased sporulation frequency compared to that of the *rstA* mutant containing the vector control (Fig. 6D), indicating that RstA is not necessary for c-di-GMP-dependent inhibition of sporulation. CD1492 and CD2492 are predicted histidine kinases that function as phosphotransfer proteins to repress sporulation and are hypothesized to directly impact Spo0A phosphorylation (28, 48). A *CD1492 CD2492* double mutant exhibited significantly increased sporulation compared to the 630Δ*erm* parent (Fig. 6A and E), and the overexpression of *dccA* in the *CD1492 CD2492* background reduced the sporulation frequency ∼3-fold (Fig. 5E), demonstrating that CD1492 and CD2492 are not a required part of the c-di-GMP signaling pathway.

We also asked whether c-di-GMP signals through SigD to control C. difficile sporulation. SigD is the flagellar alternative sigma factor that is required for both motility and efficient toxin production in C. difficile (14, 39, 49). Although a null *sigD* mutation does not affect sporulation under these conditions (comparing the 630Δ*erm* and 630Δ*erm sigD* vector control strains) (Fig. 6A and F) (39, 49), we chose to investigate the *sigD* mutant because c-di-GMP directly represses *sigD* transcription, inhibiting C. difficile motility and toxin production (13, 15, 50). The sporulation frequency was reduced only 1.9-fold when *dccA* was overexpressed in the *sigD* background, and the difference was not statistically significant (Fig. 6F). Furthermore, in the 630E background, where the phase-variable switch that controls the transcription of the flagellar operon is locked off, resulting in low *sigD* expression levels (51–53), the overexpression of *dccA* also significantly decreased sporulation by ∼20-fold (Fig. 6G). The DccA-dependent effect was not as dramatic in the 630Δ*erm sigD* background as in the other mutant backgrounds tested; however, these data altogether suggest that c-di-GMP does not primarily influence sporulation through SigD.

Finally, we tested whether c-di-GMP affects sporulation frequency through the catabolite control protein CcpA. As a transcriptional regulator, CcpA responds to glycolytic carbohydrate availability to regulate carbon and nitrogen metabolism as well as other physiological processes, including sporulation and toxin production (30, 54). The sporulation frequency was again significantly decreased by ∼20-fold when *dccA* was overexpressed in the 630E *ccpA* mutant (Fig. 6H), indicating that CcpA is not involved in c-di-GMP signaling to control sporulation initiation. Altogether, these data indicate that c-di-GMP does not significantly impact C. difficile sporulation through these known regulatory factors under the conditions tested.

## DISCUSSION

In this work, we set out to determine the impact of the bacterial second messenger c-di-GMP on C. difficile sporulation. We found that the overexpression of *dccA*, encoding a DGC that synthesizes high intracellular levels of c-di-GMP when overexpressed (13), resulted in significant decreases in early sporulation gene expression and spore formation in the epidemic R20291 and historical 630Δ*erm* strains. The conserved catalytic GGDEF motif was required for DccA-dependent inhibition of sporulation, indicating that the diguanylate cyclase synthase activity of DccA is responsible for decreased sporulation. Consistent with this result, sporulation was inhibited in a dose-dependent manner, as higher transcript levels of *dccA* coincided with fewer detected transcripts of *sigE*, which encodes an early sporulation-specific sigma factor.

Because DccA overexpression drastically altered the sporulation frequency in R20291 and 630Δ*erm*, we had anticipated a stronger sporulation phenotype in the corresponding *dccA* mutants. However, there are many additional DGCs encoded in the C. difficile genome as well as many PDEs, and these likely contribute to the intracellular concentration of c-di-GMP also, as most are catalytically active (10). Given the sheer number of encoded DGCs and that their functions may inherently exhibit redundancy, it is, in retrospect, unsurprising that the deletion of a single GGDEF domain protein does not significantly impact sporulation. Any contribution of DccA to the intracellular c-di-GMP pool may be masked by the redundant functions of similar proteins. As such, we previously found that the overexpression of DccA resulted in modest increases in the transcript levels of several encoded PDEs, suggesting that C. difficile is able to somewhat compensate for high levels of intracellular c-di-GMP (55). It is also possible that changes in c-di-GMP levels upon the loss of DccA are compensated for by the upregulation or activation of other DGCs, the downregulation or inhibition of PDEs, or both. Importantly, previous studies have shown that distinct DGCs control different c-di-GMP-regulated phenotypes, suggesting that localized pools of c-di-GMP influence only a subset of phenotypes (56, 57). It is plausible that the deletion of one or more of the other encoded DGCs in C. difficile could result in a stronger impact on the sporulation frequency. A recent report describes that the overexpression of a phosphodiesterase containing an EAL domain increases sporulation and that the deletion of that PDE decreases sporulation in C. difficile UK1, an epidemic strain that is nearly identical to R20291, corroborating our findings (58).

Interestingly, two early C. difficile sporulation regulators that are orthologous to the B. subtilis SinR transcriptional repressor regulate C. difficile intracellular c-di-GMP levels. Null mutations in the two C. difficile SinR orthologs, known as SinRR′ in R20291 and CD2214-CD2215 in 630Δ*erm*, result in increased *dccA* transcription and c-di-GMP levels and an asporogenous phenotype (20, 59). Furthermore, the deletion of *CD2214-CD2215* resulted in the differential expression of additional DGC and PDE genes in C. difficile 630Δ*erm* (20). It will be intriguing to determine if the effect of SinRR′ on C. difficile sporulation occurs through alteration of c-di-GMP levels by regulating DGC/PDE gene expression or if SinRR′ affects sporulation initiation through multiple regulatory pathways.

Identification of the c-di-GMP effector(s) that mediates c-di-GMP-dependent sporulation regulation in C. difficile is of great interest. A variety of c-di-GMP receptors that directly bind to c-di-GMP have been characterized in bacteria. These encompass a number of protein-based receptors, including proteins containing degenerate GGDEF and/or EAL domains, and two distinct types of riboswitches that alter downstream gene expression in response to c-di-GMP binding (8). C. difficile encodes a single PilZ domain protein (6), a domain that often directly binds c-di-GMP (60, 61), and a type IV pilus PilB ATPase similar to orthologs that have been shown to bind c-di-GMP (62), but there are no other known or predicted c-di-GMP protein receptors reported in C. difficile (8). C. difficile encodes at least 11 functional riboswitches that alter gene expression in response to c-di-GMP and contains 5 additional predicted riboswitches (15, 55). None of these riboswitches appear to affect the expression of sporulation-related genes; however, the conditions used in this study do not support efficient sporulation initiation in C. difficile (55). Performing transcriptomics under conditions that favor sporulation may provide insights into which regulatory pathway(s) or factor(s) is required to mediate this c-di-GMP-dependent response.

The regulatory pathway that c-di-GMP utilizes to influence sporulation remains unknown. The decrease in spore formation mediated by *dccA* overexpression does not appear to signal through CodY, CcpA, the Opp or App oligopeptide permeases, RstA, or the CD1492 and CD2492 phosphotransfer proteins. Although the effect of DccA-mediated inhibition of sporulation was slightly decreased in the *sigD* mutant, c-di-GMP is unlikely to signal solely through SigD under these conditions. Given that we know that c-di-GMP directly affects *sigD* transcription in C. difficile through the Cdi-1-3 riboswitch (13, 15, 50), it is attractive to hypothesize that SigD might have a role in sporulation. It may be possible that decreased levels of SigD are necessary for c-di-GMP to affect sporulation; in this case, testing the c-di-GMP-dependent effects on sporulation in a *sigD* mutant or in JIR8094, a phase-off, nonmotile strain with low SigD levels, may not directly answer this question. Thus far, there is no published evidence of a regulatory role for SigD in sporulation (39, 49, 55).

The impact of c-di-GMP on sporulation has been investigated in only a few other spore-forming bacteria. Interestingly, using an mCherry reporter fused to a c-di-GMP-regulated riboswitch, high c-di-GMP levels were observed in sporulating B. subtilis cells, suggesting a correlation (63), but the impact of c-di-GMP on B. subtilis sporulation has remained relatively unexplored. Studies in Bacillus thuringiensis and Bacillus anthracis found no effect on the sporulation efficiency when any of the catalytically active GGDEF/EAL/HD-GYP-encoding genes were individually deleted (64, 65). These studies in *Bacillus* sp. further underscore the differences between C. difficile and other endospore-forming bacteria in their early sporulation regulatory networks. Finally, direct c-di-GMP regulation of sporulation has been identified only in *Streptomyces* sp., where c-di-GMP inhibits spore formation directly by antagonizing the sporulation-specific sigma factor WhiG and binding directly to the transcriptional regulator BldD (66, 67). The contribution of c-di-GMP to sporulation remains an understudied field.

Utilizing c-di-GMP to inhibit sporulation may be advantageous to C. difficile, as c-di-GMP can be rapidly degraded when environmental and intracellular conditions favor sporulation. The c-di-GMP metabolic activity of a protein is often controlled by the corresponding domains encoded within the same protein. Identifying the DGCs and PDEs that affect C. difficile sporulation and investigating the function of their associated domains may reveal the environmental and intracellular signals that promote or delay sporulation. Finally, the finding that c-di-GMP is a regulator of early sporulation events in C. difficile creates new research opportunities for discovering the potentially novel regulatory pathways, c-di-GMP effectors, and molecular mechanisms that control spore formation in this significant pathogen.

## MATERIALS AND METHODS

### Bacterial strains and growth conditions.

The bacterial strains and plasmids used for this study are listed in Table 2. Clostridioides difficile was routinely cultured in brain heart infusion-supplemented (BHIS) medium in a 37°C vinyl anaerobic chamber (Coy) with an atmosphere of 5% CO_2_, 10% H_2_, and 85% N_2_ (68). C. difficile cultures were supplemented with 2 to 10 μg/ml thiamphenicol as necessary for plasmid maintenance. Escherichia coli strains were grown aerobically at 37°C in LB with 100 μg/ml ampicillin and/or 10 to 20 μg/ml chloramphenicol, and counterselection against E. coli after conjugation with C. difficile was performed using 100 μg/ml kanamycin (13).

**TABLE 2:** Bacterial strains and plasmids

Strain (lab annotation) or plasmid	Relevant genotype or feature(s)	Source and/or reference(s)
Strains		
E. coli		
HB101	F^−^ *mcrB mrr hsdS20*(r_B_^−^ m_B_^−^) *recA13 leuB6 ara-14 proA2 lacY1 galK2 xyl-5 mtl-1 rpsL20* pRK24	B. Dupuy
B. subtilis		
BS49	CU2189::Tn*916*	P. Mullany
MC1959	BS49 Tn*916*::P*_CD1421_-dccA*	This study
C. difficile		
630Δ*erm*	Erm^s^ derivative of strain 630	N. Minton; 36
R20291		35
JIR8094	Erm^s^ derivative of strain 630 (630E)	B. Dupuy; 75
JIR8094 *ccpA*::*erm*		54
RT526	R20291 pMC211	13
RT527	R20291 pDccA	13
RT539	R20291 pDccA^mut^	This study
RT762	630Δ*erm* pMC211	This study
RT763	630Δ*erm* pDccA	This study
RT764	630Δ*erm* pDccA^mut^	This study
RT993	630Δ*erm*::P*_cprA_-dccA*	41
RT1050	630Δ*erm* pP*_pilA1_*-UTR-*gusA*	41
RT1051	630Δ*erm* pP*_pilA1_*-UTR^A70G^-*gusA*	41
RT1054	630Δ*erm*::P*_cprA_-dccA* pP*_pilA1_*-UTR-*gusA*	41
RT1055	630Δ*erm*::P*_cprA_-dccA* pP*_pilA1_*-UTR^A70G^-*gusA*	41
RT1075	630Δ*erm sigD*::*erm*	39
RT2257	R20291 Δ*cmrT*	37
RT2283	R20291 Δ*cmrT* pMC211	This study
RT2284	R20291 Δ*cmrT* pDccA	This study
RT2656	R20291 Δ*dccA*	This study
RT2703	630Δ*erm* Δ*dccA*	This study
MC307	630Δ*erm oppB*::*erm appA*::*erm*	33
MC364	630Δ*erm codY*::*erm*	31
MC391	630Δ*erm rstA*::*erm*	39
MC802	630Δ*erm* Δ*CD1492 CD2492*::*erm*	This study
MC864	630Δ*erm sigD*::*erm* pMC211	This study
MC865	630Δ*erm sigD*::*erm* pDccA	This study
MC924	630Δ*erm oppB*::*erm appA*::*erm* pMC211	This study
MC925	630Δ*erm oppB*::*erm appA*::*erm* pDccA	This study
MC926	630Δ*erm rstA*::*erm* pMC211	This study
MC927	630Δ*erm rstA*::*erm* pDccA	This study
MC928	630Δ*erm* Δ*CD1492 CD2492*::*erm* pMC211	This study
MC929	630Δ*erm* Δ*CD1492 CD2492*::*erm* pDccA	This study
MC943	JIR8094 pMC211	This study
MC944	JIR8094 pDccA	This study
MC945	JIR8094 *ccpA*::*erm* pMC211	This study
MC946	JIR8094 *ccpA*::*erm* pDccA	This study
MC947	630Δ*erm codY*::*erm* pMC211	This study
MC948	630Δ*erm codY*::*erm* pDccA	This study
MC1960	630Δ*erm* Δ*dccA* Tn*916*::P*_CD1421_-dccA*	This study
MC1961	R20291 Δ*dccA* Tn*916*::P*_CD1421_-dccA*	This study

Plasmids		
pRK24	Tra^+^ Mob^+^; *bla tet*	76
pSMB47	Tn*916* integrational vector; *catP erm*	77
pCR2.1	*bla kan*	Invitrogen
pMSR	Allelic exchange in C. difficile 630	42
pMSR0	Allelic exchange in C. difficile R20291	42
pMSR::Δ*dccA*	*dccA* deletion construct in pMSR	This study
pMSR0::Δ*dccA*	*dccA* deletion construct in pMSR0	This study
pCE240	C. difficile Targetron construct based on pJIR750ai (group II intron; *ermB*::*RAM ltrA*); *catP*	C. Ellermeier
pMC330	pCR2.1 with group II intron targeted to *CD2492*	This study
pMC333	pCE240 with *CD2492*-targeted intron	This study
pMC336	pMC123 with *CD2492*-targeted intron	This study
pMC211	E. coli*-*C. difficile shuttle vector; *bla catP*	13, 33
pDccA	*CD1420* from 630 in pMC211	13
pDccA^mut^	*CD1420* (AADEF) in pMC211	13
pMC1094	P*_CD1421_-dccA* in pSMB47	This study
pP*_pilA1_*-UTR-*gusA*		41
pP*_pilA1_*-UTR^A70G^-*gusA*		41

### Strain and plasmid construction.

C. difficile strains 630 (GenBank accession no. NC_009089.1) and R20291 (GenBank accession no. FN545816.1) were used as the basis for primer design and PCR amplification (oligonucleotides used in this study are listed in Table 3). The *dccA* mutants were constructed using the pseudo-suicide vectors pMSR and pMSR0 (42). Upstream and downstream homology regions were amplified from the 630Δ*erm* genome with primer pairs R2928/R2929 and R2930/R2931, respectively. The fragments were Gibson assembled (New England BioLabs [NEB]) into SalI/XhoI-digested pMSR to create pMSR::Δ*dccA*. Similar fragments were amplified from the R20291 chromosome using the same primers and assembled into pMSR0 to create pMSR0::Δ*dccA*. Chloramphenicol-resistant colonies were confirmed by PCR with plasmid-specific primer pair R838/R1832 (pMSR) or R2743/R2744 (pMSR0).

**TABLE 3:** Oligonucleotides

Primer	Sequence (5′→3′)	Reference, source, or use (reference)
CD1420qF	5′-AAGAAACTCCCTGATAATATTGCTAA	13
CD1420qR	5′-ACATTCCAATAGCTTGTAGTATCTTT	13
EBSu	5′-CGAAATTAGAAACTTGCGTTCAGTAAAC	Sigma-Aldrich
oMC44	5′-CTAGCTGCTCCTATGTCTCACATC	Forward primer for *rpoC* (34)
oMC45	5′-CCAGTCTCTCCTGGATCAACTA	Reverse primer for *rpoC* (34)
oMC309	5′-GGAGAATACAGAGATTTGATTGATTC	Forward primer for *CD2492*::*erm* verification
oMC317	5′-AAAAGCTTTTGCAACCCACGTCGATCGTGAAGTGATCTTAATCGTGCGCCCAGATAGGGTG	IBS for *CD2492*-targeted intron
oMC318	5′-CAGATTGTACAAATGTGGTGATAACAGATAAGTCTTAATCTCTAACTTACCTTTCTTTGT	EBS1 for *CD2492*-targeted intron
oMC319	5′-CGCAAGTTTCTAATTTCGATTATCACTCGATAGAGGAAAGTGTCT	EBS2 for *CD2492*-targeted intron
oMC338	5′-TCCCATTTGCCTTTATTTGAACTTGA	Reverse primer for *CD2492*::*erm* verification (39)
oMC339	5′-GGGCAAATATACTTCCTCCTCCAT	Forward primer for *sigE* (*CD2643*) (33)
oMC340	5′-TGACTTTACACTTTCATCTGTTTCTAGC	Reverse primer for *sigE* (*CD2643*) (33)
oMC2910	5′-GACCACACCCGTCCTGTGGATCCCCATCTCTATGTAATATTTTTCATATTAAAACTGATTTC	Forward primer for P*CD1421*
oMC2911	5′-CTTTAAACATATTAATTTCTCCAAAATAAAATACTTTGTACTGGTATTCCTCCATAAGATACTTTAAATTTTG	Reverse primer for P*CD1421*
oMC2912	5′-CAAAATTTAAAGTATCTTATGGAGGAATACCAGTACAAAGTATTTTATTTTGGAGAAATTAATATGTTTAAAG	Forward primer for *dccA* (*CD1420*)
oMC2913	5′-CCGCCGCAAGGAATGGTGCATGCTTAATAATCATTTTTATCAAATTTTTTCTTGTTTTTCTCC	Reverse primer for *dccA* (*CD1420*)
R838	5′-GTAAAACGACGGCCAGT	Reverse screening primer for pMSR
R1832	5′-TATTTCGATGCCCTGGACT	Forward screening primer for pMSR
R2743	5′-GTGTTATCAATTGCACTACTCATGG	Forward screening primer for pMSR0
R2744	5′-GTTGAACCATTAGCTAAGGATTCAG	Reverse screening primer for pMSR0
R2926	5′-CTGATATAGGAAAATCTTTAATAGAGAAG	Forward screening primer for *dccA* chromosomal deletion
R2927	5′-TCCATGAACTCATCATATGTGTATCC	Reverse screening primer for *dccA* chromosomal deletion
R2928	5′-TTCGGATCCTCTAGAGTCGACTGCAAGATATGAAAAAACTAAGAGC	Forward primer for upstream fragment of *dccA* deletion construct
R2929	5′-CAAATTTTTTCTTGTTTTTCTCCATATTAATTTCTCCAAAATAAAATACTTTGTACTAG	Reverse primer for upstream fragment of *dccA* deletion construct
R2930	5′-GTATTTTATTTTGGAGAAATTAATATGGAGAAAAACAAGAAAAAATTTGATAAAAATG	Forward primer for downstream fragment of *dccA* deletion construct
R2931	5′-ATGTCTGCAGGCCTCGAGGAGTATGTACTATCATCATTGCTACC	Reverse primer for downstream fragment of *dccA* deletion construct

**FIG 7:**
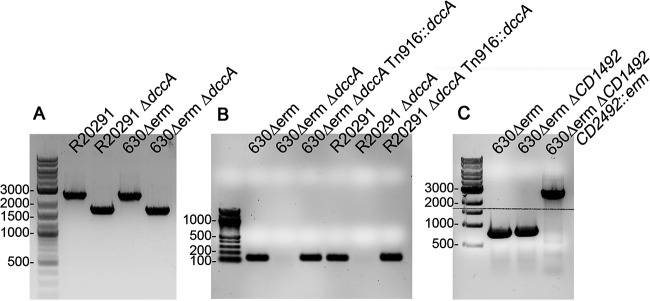
PCR verification of the constructed strains. (A) PCR confirmation of Δ*dccA* in the R20291 and 630Δ*erm* backgrounds using primer pair R2926/R2927. Shown are strains R20291, R20291 Δ*dccA* (RT2656), 630Δ*erm*, and 630Δ*erm* Δ*dccA* (RT2703). The expected PCR products’ sizes are 2.5 kb for the wild-type allele and 1.7 kb for the deletion mutants. (B) PCR verification of Tn*916*::P*_CD1421_-dccA* chromosomal integration in 630Δ*erm* and R20291 using the internal qRT-PCR *dccA* primer pair CD1420qF/CD1420qR. Shown are strains 630Δ*erm* Δ*dccA* (RT2703), 630Δ*erm* Δ*dccA* Tn*916*::P*_CD1421_-dccA* (MC1960), R20291, R20291 Δ*dccA* (RT2656), and R20291 Δ*dccA* Tn*916*::P*_CD1421_-dccA* (MC1961). The expected PCR product size is 140 bp. (C) PCR confirmation of the *CD2492*-targeted intron in the 630Δ*erm* Δ*CD1492* background (MC674). Shown are strains 630Δ*erm*, 630Δ*erm* Δ*CD1492* (MC674), and 630Δ*erm* Δ*CD1492 CD2492*::*erm* (MC802) using primer pair oMC309/oMC338. The expected PCR products’ sizes are 811 bp for the wild-type *CD2492* allele and ∼2,811 bp for *CD2492*::*erm*.

To create the *dccA* mutants, the pMSR::Δ*dccA* and pMSR0::Δ*dccA* plasmids were transformed into E. coli HB101 pRK24 for conjugation with C. difficile 630Δ*erm* and R20291, respectively. Subsequent steps were done essentially as previously described (42). Briefly, large thiamphenicol- and kanamycin-resistant colonies, which presumably had integrated the plasmid into the chromosome to allow for more rapid growth, were streaked again on BHIS medium with 10 μg/ml thiamphenicol and 100 μg/ml kanamycin to ensure purity. Next, large colonies were streaked onto BHIS medium with 100 ng/ml anhydrotetracycline (ATc) to induce the expression of the toxin gene and eliminate bacteria that still contained the vector. Colonies were screened for the 0.8-kb deletion of *dccA* using primer pair R2926/R2927 (Fig. 7A). Genomic DNA was isolated from potential mutants, and the *dccA* region was PCR amplified using primer pair R2926/R2927 and sequenced to confirm the integrity of the sequence.

The 630Δ*erm* and R20291 *dccA* mutants were complemented using a Bacillus subtilis donor strain, BS49, carrying the conjugative transposon Tn*916* to transfer the *dccA* gene driven by its native promoter, which is encoded upstream of *CD1421* (MC1959). To create the plasmid carrying the Tn*916*::P*_CD1421_-dccA* construct (pMC1094), P*_CD1421_* and *dccA* were spliced by overlapping PCR using primer pairs oMC2910/2911 and oMC2912/2913 and Gibson assembled into BamHI/SphI-digested pSMB47. Erythromycin-resistant colonies were confirmed by PCR with primer pair CD1420qF/CD1420qR (Fig. 7B).

To create the 630Δ*erm* Δ*CD1492 CD2492*::*erm* double mutant, the Targetron-based group II intron from pCE240 was retargeted using the targeting site reported previously by Underwood et al. to create pMC336 (69). Briefly, the *CD2492*-targeted intron was amplified using primers oMC317, oMC318, oMC319, and EBSu and TA cloned into pCR2.1 to create pMC330. A BsrGI/HindIII-digested fragment containing the *CD2492-*targeted intron was subcloned into pCE240 to create pMC333. Finally, an SphI/SfoI-digested fragment containing the *CD2492-*targeted intron was subcloned into pMC123 to create pMC336. The resulting construct, pMC336, was conjugated into the 630Δ*erm CD1492* strain (MC674), and erythromycin-resistant mutants were screened for the 2-kb Targetron insertion within *CD2492* using primer pair oMC309/338 (Fig. 7C). Notably, the targeting site was not located in the 254a site within the CD2492 coding region noted by Underwood et al. (69) but rather was located at 318s (data not shown).

### Sporulation assays.

C. difficile strains were grown overnight in BHIS medium supplemented with 0.1% taurocholate and 0.2% fructose to aid in spore germination and prevent spore formation, respectively (70, 71). Thiamphenicol (5 μg/ml) was included for plasmid maintenance when necessary. When strains reached mid-exponential phase (optical density at 600 nm [OD_600_] of ∼0.5), 250-μl aliquots were applied to the surface of 70:30 agar containing 2 μg/ml thiamphenicol and 0 to 0.5 μg/ml nisin (71). After 24 h of growth, ethanol-resistant sporulation assays were performed as previously described (48, 72). Briefly, cells were scraped from the plate surface and suspended in BHIS medium to an OD_600_ of ∼1. To enumerate vegetative cells, the cell suspensions were serially diluted in BHIS medium and plated onto BHIS plates. Simultaneously, 0.5-ml aliquots of the cell suspensions were mixed thoroughly with 0.3 ml 95% ethanol and 0.2 ml distilled water (dH_2_O) (final concentration, 28.5% ethanol) and incubated for 15 min to eliminate all vegetative cells. Ethanol-treated cells were then serially diluted in 1× phosphate-buffered saline (PBS) with 0.1% taurocholate and plated onto BHIS medium supplemented with 0.1% taurocholate. Total CFU were enumerated after at least 24 h of growth, and the sporulation frequency was calculated as the number of ethanol-resistant spores divided by the total number of vegetative and ethanol-resistant spores combined. A *spo0A* mutant (MC310) was used as a negative control in all assays.

### Phase-contrast microscopy.

Phase-contrast microscopy was performed at H_24_ as described previously (33). Briefly, cells were concentrated by pelleting 0.5 ml of the cell suspension, and the concentrated cell suspension was applied to a 0.7% agarose pad on a slide. Cells were imaged with a 100× Ph3 oil immersion objective on a Nikon Eclipse Ci-L microscope. At least two fields of view were captured with a DS-Fi2 camera from at least three independent experiments for each strain tested.

### Quantitative reverse transcription-PCR analysis.

RNA was isolated from C. difficile strains grown on 70:30 sporulation agar at H_12_ and DNase I treated as previously described (13, 29, 33, 34). cDNA was synthesized using random hexamers, and quantitative real-time-PCRs were performed in triplicate (Bioline) and monitored using a Roche LightCycler 96 system. The *rpoC* transcript (primer pair oMC44/oMC45) was used as the reference gene (34). Controls with no reverse transcriptase were included for all templates and primer sets. The data were analyzed by the 2^−ΔΔ^*^CT^* method (73), with normalization to *rpoC* and the indicated reference condition or strain. The results represent the means and standard errors of the means from at least three independent experiments.

### Western blotting.

The indicated strains were grown on 70:30 sporulation agar supplemented with 2 μg/ml thiamphenicol and 0.5 μg/ml nisin and harvested at H_12_. Total protein from the cell lysates was quantitated using the Pierce Micro bicinchoninic acid (BCA) protein assay kit (Thermo Scientific), 2.5 μg of total protein was separated by electrophoresis on a precast 4 to 20% TGX stain-free gradient gel (Bio-Rad), and total protein was imaged using a ChemiDoc system (Bio-Rad). Protein was transferred to a 0.45-μm nitrocellulose membrane, and Western blot analysis was performed with mouse anti-Spo0A (71) as the primary antibody and goat anti-mouse conjugated with Alexa Fluor 488 (Invitrogen) as the secondary antibody. Imaging and densitometry were performed with a ChemiDoc system and Image Lab software (Bio-Rad), respectively, for three independent experiments.

### β-Glucuronidase reporter assays.

β-Glucuronidase assays were performed as previously detailed (41, 74). Briefly, strains were grown on 70:30 sporulation agar as indicated above and harvested at H_12_ and H_15_ by scraping the plates and suspending the cells in BHIS medium to an OD_600_ of ∼0.5 to 0.7. Two 1-ml aliquots were pelleted and stored at −20°C overnight. The pellets were suspended in 0.8 ml Z buffer and 0.05 ml 0.01% SDS. The samples were vortexed, incubated at 37°C for 5 min, and then chilled on ice for 5 min. After a 1-min incubation at 37°C to warm the samples up to room temperature, the enzymatic reaction was started with the addition of 100 μl of 40 μg/ml *p*-nitrophenol-β-d-glucuronide and stopped with 0.4 ml 1 M Na_2_CO_3_. Cell debris was pelleted, and the *A*_420_ and *A*_550_ were measured using a BioTek Synergy H1 plate reader. Specific activity was normalized by the OD_600_. Three independent biological replicates were used to calculate the means and standard errors of the means.
